# Screening for Sarcopenia among Elderly Arab Females: Influence of Body Composition, Lifestyle, Irisin, and Vitamin D

**DOI:** 10.3390/nu14091855

**Published:** 2022-04-29

**Authors:** Tafany A. Alsaawi, Dara Aldisi, Mahmoud M. A. Abulmeaty, Malak N. K. Khattak, Abdullah M. Alnaami, Shaun Sabico, Nasser M. Al-Daghri

**Affiliations:** 1 Department of Community Health Sciences, College of Applied Medical Sciences, King Saud University, Riyadh 11362, Saudi Arabia; tafanyalsaawi@gmail.com (T.A.A.); daldisi@ksu.edu.sa (D.A.); mabulmeaty@ksu.edu.sa (M.M.A.A.); 2 Chair for Biomarkers of Chronic Diseases, Biochemistry Department, College of Science, King Saud University, Riyadh 11451, Saudi Arabia; malaknawaz@yahoo.com (M.N.K.K.); aalnaami@yahoo.com (A.M.A.); ssabico@ksu.edu.sa (S.S.)

**Keywords:** diet, geriatric, lifestyle, physical activity, sarcopenia

## Abstract

Sarcopenia is the loss of skeletal muscle mass, and is most common in older people. The present multi-center cross-sectional study aimed to determine the prevalence of sarcopenia and possible risk factors among Arab elderly females. A total of 131 ambulatory Saudi elderly females aged 60–85 years (mean age 65.9 ± 5.5 years) were recruited to participate. A general questionnaire with questions related to sociodemographic factors, medical history, diet, physical activity, and lifestyle was administered. Anthropometrics and muscle assessments were done. Fasting blood glucose and lipids were measured routinely. Circulating 25(OH)D and irisin levels were measured using commercially available assays. Sarcopenia was assessed using the criteria of the Asian Working Group for Sarcopenia (AWGS). Over-all prevalence of sarcopenia was 19.8% (26 out of 131 participants). Novel measures such as abdominal volume index (AVI), dietary fiber, and irisin were found to be significantly lower in the sarcopenia group than those without sarcopenia, independent of age. No associations were found with physical activity or dietary and lifestyle habits. In conclusion, sarcopenia is relatively common among Arab elderly females. Longitudinal studies are needed to determine whether lifestyle modifications can decrease the incidence of sarcopenia in this population. Irisin maybe a promising biomarker for sarcopenia but needs to be confirmed using larger sample sizes.

## 1. Introduction

Sarcopenia is the gradual and general loss of skeletal muscle mass and strength associated with aging which might lead to serious adverse outcomes such as physical disability and poor quality of life [1]. In 2010, the European Working Group on Sarcopenia in Older People (EWGSOP) established an operational definition and diagnostic criteria for sarcopenia [1]. Subsequently, the Asian Working Group on Sarcopenia (AWGS) adapted its own definition to describe sarcopenia [2]. In 2018, the EWGSOP considered sarcopenia as a muscle disease (muscle failure) [3]. Many factors lead to the progression of sarcopenia and these can contribute to the severity and staging of the reduction in muscle mass, strength, and performance [4]. While older age might be the most important among the reported risk factors, other determinants such as marital status, lifestyle, physical inactivity, poor dietary intake, and diseases (osteoporosis, metabolic diseases, etc.), were also observed to be associated with sarcopenia [5]. Since sarcopenia is a multifactorial syndrome, numerous biomarkers for sarcopenia have been investigated to better understand the different pathophysiologic mechanisms associated with it [6].

Vitamin D level has a well-known effect on muscle performance as it binds to the nuclear vitamin D receptor (VDR) on muscle fibers which leads to an increase in size and hence, improved muscle strength [7,8,9]. Moreover, a recently discovered myokine known as irisin was found to be a strong predictor for sarcopenia [10,11,12,13]. A significant increase or decrease in muscle mass and function from prolonged shifts in muscle protein anabolism, catabolism, or the combination of both processes are controlled by numerous stimuli including physical activity and dietary intake [14]. Furthermore, several studies suggest that with sarcopenia, there is an association between dietary protein intake and physical activity [15,16,17,18,19,20,21,22,23,24]. A systematic review conducted in 37 randomized controlled trials showed that muscle mass and physical activity increased after protein supplementation and exercise interventions [25]. Many studies also suggested the use of anthropometric measurements for the screening of sarcopenia as they were strongly associated with muscle mass strength and performance [26,27,28,29,30,31,32,33].

Despite a surge in sarcopenia research among nations with growing elderly populations, there is scarcity of observational studies in Saudi Arabia and the Middle East in general. To date, studies have been limited to epidemiology and health outcomes, mostly in men [34,35,36,37]. In order to fill this gap, the present study aimed to determine, for the first time in an Arabian elderly female population, the association of dietary intake as well as known markers for musculoskeletal strength such as irisin and vitamin D with sarcopenia.

## 2. Materials and Methods

### 2.1. Study Design and Participants

In this multi-center cross-sectional study, Saudi females aged 60–85 years with or without sarcopenia were recruited from primary health-care centers (Aldiriyah and Alsalam centers), in addition to community centers (King Salman Social Center and Quran Memorizing Centers) in Riyadh city, Saudi Arabia, from March 2019 to December 2019. Participants who required a cane, wheelchair, or other assistance tools, had artificial limbs or a history of chronic obstructive pulmonary disorder (COPD), congestive heart failure (CHF), chronic renal failure (CRF), active cancer, or cirrhosis liver failure, or had poorly controlled medical problems or refused to participate were excluded. The study was conducted in accordance with the Declaration of Helsinki and was approved by the College of Medicine Institutional Review Board (IRB) in King Saud University, Riyadh, Saudi Arabia (No.19/0300/IRB) as well as by the Ministry of Health (IRB NO.2019-0043E). Written informed consent was provided by all participants prior to inclusion. For the purpose of the present study, participants were stratified according to sarcopenia status. Participants had sarcopenia if they had low muscle mass (muscle mass < 5.7 kg/m^2^) in addition to low muscle strength (handgrip strength < 18 kg) or low physical performance (TUG < 20 s). Severe sarcopenia was considered if all three criteria were present [2].

### 2.2. Demographic and Lifestyle Assessment

Demographic data, such as socioeconomic status and medical history were taken from participants through a general questionnaire duly administered by the investigators in designated primary-care centers. Furthermore, a food frequency questionnaire was administered by a certified dietitian to assess macronutrient intake and this was analyzed using Esha food processor software (version 11.7, Esha Research, Salem, OR, USA) [38]. Lifestyle habits and physical activity levels were assessed using the same questionnaire [38].

### 2.3. Anthropometric and Body Composition Measurements

Anthropometric measurements were carried out by the investigators for all participants. Each participant was asked to stand barefoot on a stadiometer to measure height (cm) to the nearest 0.1 cm followed by a bioelectrical impedance analysis (BIA) (Tanita BC-418, Tanita Co, Tokyo, Japan) to measure weight (kg) to the nearest 0.1 kg. Body mass index (kg/m^2^) was calculated. Waist circumference (WC) and hip circumference (HC) were measured using a standard tape measure. The mid-arm muscle area (MAMA), mid-arm circumference (MAC), and triceps skinfold-thickness were calculated. The conicity index was determined using the formula (CI = WC (m)/[0.109 × √{weight (kg)/Height (m)}] [39]. The abdominal volume index (AVI) was calculated accordingly [AVI = [2 × (WC)^2^ + 0.7 × (waist–hip)^2^]/1000] [40].

### 2.4. Muscle Mass, Strength, and Performance

Bioelectrical impedance analysis (BIA) (Tanita BC-418, Tanita Co, Japan) was used to determine body composition for each participant. Participants with a muscle mass < 6.4 kg/m^2^ were considered to have low muscle mass according to the AWGS [2]. Handgrip strength (HS) (Lafayette hydraulic hand dynamometer, USA) was used to measure muscle strength by asking participants to squeeze the hydraulic dynamometer with the right and left hand and the average measure was taken. Low handgrip strength is suggested to be defined as <18 kg for women by the AWGS [2]. Participants’ muscle performance was assessed using a 3 m timed up-and-go test (TUG). Each participant was asked to sit in a chair then rise and walk at normal speed for 3 m and go back to the same seat. The time it took to accomplish the test was recorded, with less than 20 s considered as low muscle performance based on EWGSOP recommendations [1]. All measurements were assessed with the participant standing and in a non-fasting state.

### 2.5. Biochemical Analysis

Five milliliters (5 mL) of fasting blood sample were drawn from each participant by a registered nurse prior to muscle strength assessment. Obtained samples were used to assess fasting glucose and lipid profile using an automated biochemical analyzer (Konelab, Espoo, Finland). Blood samples were centrifuged (3000 RPM for 10 min) then, stored in a −80 °C freezer before the analysis. Total serum 25(OH)D was measured using commercial electrochemiluminescence immunoassay. Intra- and inter-assay coefficients of variations were 4.6% and 5.3%, respectively (Roche Diagnostics, Penzberg, Germany), while commercially available assay (Biovendor, Karasek, Czech Republic) was used to assess circulating irisin levels (intra- and inter-assay coefficients of variations were 6.9% and 9%, respectively), as performed in previous investigations [41,42]. All biochemical analyses were performed in the Chair for Biomarkers of Chronic Diseases (CBCD), King Saud University, Riyadh, Saudi Arabia.

### 2.6. Statistical Analysis

The sample size was derived based on the protective odds against sarcopenia among individuals engaged in high-level activities (OR, 0.29; 95% CI, 0.15–0.56) [22]. The required sample size was N = 127, given alpha = 0.01. Statistical analysis was done using the Statistical Package for Social Sciences (version 25, SPSS) software. Categorical variables were shown as frequency and percentages (%), while continuous variables were shown as mean ± standard deviation (SD). The chi-square test was used to compare differences between sociodemographic factors and medical history. The independent sample T-test was used to compare continuous variables between groups. Binary logistic regression was used to independently assess the factors associated with sarcopenia. Post-hoc power calculation was done using G*power and showed 88.3% actual power using the irisin mean level (effect size = 0.459 with sample sizes n1 = 26 and n2 = 105). Significance was set at *p* < 0.05.

## 3. Results

### 3.1. Participant Characteristics

The study population included 131 Saudi females with a mean age of 65.9 ± 5.5 years. Twenty-six of the participants had sarcopenia (prevalence of 19.8%). The majority of the study participants were married (65.9%), illiterate (52.3%), and unemployed (88.6%). The majority of the participants were obese (61.8%) and obesity was significantly more common in the non-sarcopenia group than in the sarcopenia group (70.5% versus 27%; *p* < 0.001). No differences were observed for the rest of the comorbidities with the exception of hypothyroidism, where all cases were found in the non-sarcopenia group (*p* = 0.02) (Table 1).

The majority of the participants (77%) were not engaged in any type of physical activity (Supplementary Table S1) with no difference being observed between groups. No differences were also observed in lifestyle behaviors in terms of sleeping patterns and sub exposure (Supplementary Table S2).

### 3.2. Clinical Differences among Participants with and without Sarcopenia

Table 2 shows the clinical differences of participants with and without sarcopenia. Significantly lower indices in the sarcopenia group were observed with respect to BMI and waist and hip circumference as well as MAC, MAMA, and AVI (all *p*-values <0.001) as compared to those without sarcopenia. Furthermore, and as expected, all the indices for muscle mass, strength, and performance were significantly lower in the sarcopenia group than the non-sarcopenia group with the exception of TUG (*p* = 0.53). Lastly, circulating irisin was significantly lower in the sarcopenia group than in the non-sarcopenia group (*p* = 0.001).

### 3.3. Factors Associated with Sarcopenia

Table 3 shows anthropometric factors related to sarcopenia using bivariate logistic regression analysis. Participants with high BMI were less likely to have sarcopenia (OR = 0.79; 95% CI, 0.71–0.89; *p* < 0.001), Similarly, high waist circumference and hip circumference decreased the odds of sarcopenia (OR = 0.91; 95% CI, 0.86–0.96; *p* < 0.001). Furthermore, high mid-arm circumference (OR = 0.75, 95% CI: 0.64–0.87; *p* < 0.001), and high mid-arm muscle area (OR = 0.90; 95% CI, 0.85–0.95; *p* < 0.001) were significantly associated with decreased risk of sarcopenia. High abdominal volume index was found to decrease the odds of sarcopenia by 21% (OR = 0.79; 95% CI, 0.69–0.91; *p* = 0.001). Among the biochemical parameters assessed, low irisin was associated with sarcopenia (OR = 0.97; 95% CI, 0.95–0.99; *p* = 0.002). The rest of the biochemical markers analyzed in this study were not found to be correlated with sarcopenia. Lastly, among the macronutrient intake, only low total fiber intake was associated with sarcopenia (OR = 0.94; 95% CI, 0.88–0.99; *p* = 0.03) (Table 3).

**Figure 1:**
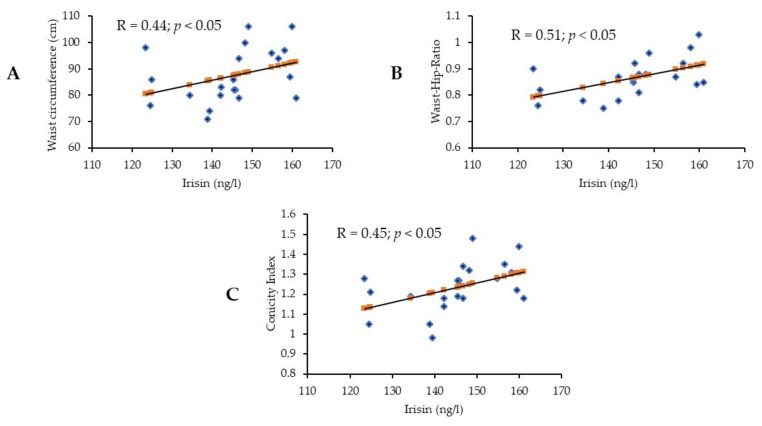
Significant positive associations of irisin with (**A**) waist circumference, (**B**) waist to hip ratio, and (**C**) conicity index in the sarcopenia group.

Lastly, bivariate associations showed a significant positive correlation between irisin level and waist circumference (r = 0.44, *p* = 0.05) (Figure 1A) as well as a significant positive correlation between waist to hip ratio (Figure 1B) and conicity index (Figure 1C) (r = 0.51, *p* = 0.05; and r = 0.45, *p* = 0.05, respectively), only in the sarcopenia group.

## 4. Discussion

The present study attempted to determine the associations of sarcopenia to several factors among elderly Arab females with or without the condition and the differences between these factors. To the best of our knowledge, the present study is the first of its kind to investigate these associations among Arab elderly females. The main findings of the present investigation included the high prevalence of sarcopenia (19.8%) among elderly Arab women, the significantly lower irisin levels among those with sarcopenia, and the significant positive associations of irisin with body composition measures observed only among those with sarcopenia.

Consistent with our results, several studies showed a strong association between anthropometric measures and sarcopenia [29,33,39]. Our findings are supported by a cross-sectional study that reported an inverse association of BMI and WC with sarcopenia; these factors were considered predictors of sarcopenia among elders in the Amazon region [29]. Additionally, a cross-sectional study observed that low BMI in Singaporean elders was strongly correlated with sarcopenia, aside from high WC [28]. On the other hand, BMI was observed to be a determinant of sarcopenia with a comparable risk factor to low physical performance among older adults with diabetes in Japan [27]. Moreover, a prospective cohort study stated that MAC was considered as the best anthropometric measure associated with sarcopenia [31]. Likewise, a multi-ethnic cross-sectional study suggested that high HC was associated with low sarcopenia risk in older Asians [30]. Obese individuals could also have sarcopenia (sarcopenic obesity) if such individuals experience muscle-mass loss with subsequent increase in adiposity [1,27].

Advance loss of skeletal muscle mass occurs with aging and has been linked to impaired skeletal muscle protein synthesis, caused by reduced amino acid delivery to aged skeletal muscle [43]. Consequently, protein intake was found to be strongly associated with sarcopenia [15]. Our findings in contrast found no significant correlation between protein intake and sarcopenia, which was similar to a cross-sectional study which found no difference between protein intake and handgrip strength among elderly women who consumed higher levels of protein [44]. Even though dietary fiber was significantly low in the sarcopenia group and remained significant after using logistic regression, no comparable literature was found to support this finding. Nevertheless, many studies found a strong relationship between the Mediterranean diet, which is high in fiber, and reduced risk of sarcopenia and frailty in older adults [45,46].

Physical activity was not correlated with sarcopenia in the present study, consistent with the findings observed among Chinese elders which also found no relationship between sarcopenia and physical activity [47]. Most of our study participants were not engaged in any physical activity—only 22.1% were engaged in light physical activity, 19% were engaged in moderate activities, and none in vigorous activities. Thus, this low percentage of the population engaged in physical activities might explain our results, in addition to the variety of the assessment methods that have been used in previous studies.

Irisin is a myokine that is proteolytically cleaved and secreted from the fibronectin type III domain-containing protein 5 and primarily secreted in the skeletal muscle [48,49]. Therefore, several studies have investigated the association of irisin with muscle mass and strength [10,11,12,13]. In our study, the sarcopenia group had significantly lower irisin levels than the non-sarcopenia group (*p* = 0.001). Moreover, high irisin was associated with lower odds of sarcopenia (*p* = 0.002). Our results are consistent with a previous cross-sectional study in South Korea that found that serum irisin levels were significantly lower in postmenopausal females diagnosed with sarcopenia compared to those without [11], but also contradicts a more recent observation, also among South Koreans, that irisin has no association between clinical muscle parameters [50]. This discrepancy in findings within the same population may be due to sample size issues and the assays used, as well as ethnic differences with respect to the present findings.

In the current study, we reported that irisin had a significant positive correlation with WC, WHR, and CI in the sarcopenia group. Similarly, a cross-sectional study conducted among 151 Caucasian and African American males and females aged > 35 years reported that irisin was positively associated with WC and WHR in both genders [51]. On the other hand, 1115 obese Chinese adults with a mean age of 53.2 + 7.2 years were enrolled in a cross-sectional study that revealed that irisin level was inversely associated with waist circumference [52]. A cohort study that included 76 middle-aged Caucasian men showed that irisin was inversely correlated with WHR [53]. Although irisin is mostly known as a myokine, it is also released from adipose tissue, which can partially explain its association with indicators of obesity [49,54,55,56,57,58,59].

Our data revealed that vitamin D had no significant correlation with sarcopenia. This is comparable to a similar case-control study which found no difference in the mean serum 25(OH)D of British elders with and without sarcopenia [60]. In contrast, a cross-sectional study conducted among Dutch elderly subjects found that 25(OH)D was significantly lower in sarcopenic subjects than in non-sarcopenic subjects [61]. The inconsistencies between our findings and the studies supporting the association between vitamin D and sarcopenia might be due to supplement use and the season of blood sampling. In Saudi Arabia and the Gulf Cooperation Council (GCC) countries, regional guidelines promote vitamin D supplementation of up to 2000 IU per day among postmenopausal women [62,63], which explains why the mean vitamin D status for both groups in the present study are within the sufficient level. Further studies may be required, particularly intervention trials, to determine whether vitamin D status correction confers beneficial effects among elders with sarcopenia.

The authors acknowledge some limitations. First, this was a cross-sectional study thus causality could not be assessed. The lack of bone mineral density assessment limited the study’s ability to determine those with possible osteosarcopenia, which is also impacted by both physical activity and nutrition [64]. Lastly, the small sample size and the female exclusivity of the population used limits the generalizability of findings.

## 5. Conclusions

Sarcopenia is common among elderly Arab females with a multi-causal etiology and many risk factors. Novel measures such as abdominal volume index, dietary fiber, and irisin were found to be significantly lower among those with sarcopenia than those without. Moreover, irisin levels were significantly associated with abdominal obesity among those with sarcopenia. Despite the lack of association between sarcopenia, vitamin D, physical activity, and lifestyle in this population, findings should be further explored prospectively to determine whether lifestyle modifications through nutrition, supplementation, and exercise can decrease the incidence of sarcopenia among elderly Arab females.

## Figures and Tables

**Table 1:** General characteristics of participants according to sarcopenia status.

Parameters	All	Non-Sarcopenia	Sarcopenia	*p*-Value
N	131	105	26	
Age (years)	65.9 ± 5.5	65.5 ± 5.4	67.5 ± 5.7	0.11
Education				
Illiterate	69 (52.3)	55 (51.9)	14 (53.8)	0.86
Elementary	26 (19.7)	19 (17.9)	7 (26.9)	
Middle school	13 (9.8)	11 (10.4)	2 (7.7)	
High School	8 (6.1)	7 (6.6)	1 (3.8)	
College degree	15 (11.4)	13 (12.3)	2 (7.7)	
Postgraduate	1 (0.8)	1 (0.9)	0 (0.0)	
Marital Status				
Married	87 (65.9)	71 (67.0)	16 (61.5)	0.63
Widowed	43 (32.6)	33 (31.1)	10 (38.5)	
Divorced	2 (1.5)	2 (1.9)	0 (0.0)	
Employment				
None	117 (88.6)	94 (88.7)	23 (88.5)	0.75
Retired	13 (9.8)	10 (9.4)	3 (11.5)	
Home Business	2 (1.5)	2 (1.9)	0 (0.0)	
Medical history				
Obesity	81 (61.8)	74 (70.5)	7 (27)	<0.001
Type 2 diabetes	78 (59.5)	61 (58.1)	17 (65.4)	0.33
Hypertension	84 (35.9)	66 (62.9)	18 (69.2)	0.36
High cholesterol	55 (42.0)	45 (42.9)	10 (38.5)	0.43
Osteoporosis	9 (6.9)	7 (6.7)	2 (7.7)	0.86
Rheumatoid arthritis	7 (5.3)	6 (5.7)	1 (3.8)	0.70
Asthma	10 (7.6)	9 (8.6)	1 (3.8)	0.42
Hypothyroidism	16 (12.2)	16 (15.2)	0 (0.0)	0.02
Comorbidity	89 (67.9)	72 (68.6)	17 (65.4)	0.46

**Note**: Data presented as mean ± SD, N (%).

**Table 2:** Clinical characteristics of participants according to sarcopenia status.

Anthropometrics	All	Non-Sarcopenia	Sarcopenia	*p*-Value
N	131	105	26	
BMI (kg/m^2^)	31.9 ± 5.4	32.9 ± 5.3	27.8 ± 2.7	<0.001
Waist (cm)	95.8 ± 11.7	97.9 ± 11.2	87.5 ± 9.7	<0.001
Hips (cm)	111.1 ± 12.4	113.2 ± 12.7	102.7 ± 6.6	<0.001
WHR	0.86 ± 0.07	0.86 ± 0.08	0.86 ± 0.07	0.82
MAC	29.5 ± 4.6	30.3 ± 4.5	26.2 ± 3.1	<0.001
TSF	17.7 ± 3.6	17.9 ± 3.6	16.8 ± 3.2	0.16
CI	1.2 ± 0.1	1.3 ± 0.10	1.2 ± 0.1	0.48
MAMA	43.6 ± 11.4	45.8 ± 11.0	35.3 ± 8.5	<0.001
AVI	18.4 ± 4.5	19.2 ± 4.4	15.5 ± 3.4	<0.001
**Muscle Mass, Strength, and Performance**				
Muscle mass	41.1 ± 5.2	42.4 ± 4.8	35.9 ± 2.8	<0.001
Right leg muscle	6.9 ± 1.1	7.2 ± 1.0	6.1 ± 0.7	<0.001
Left leg muscle	7.0 ± 1.1	7.2 ± 1.0	6.4 ± 1.4	0.002
Right arm muscle	2.0 ± 0.3	2.1 ± 0.3	1.7 ± 0.2	<0.001
Left arm muscle	2.1 ± 0.3	2.2 ± 0.3	1.8 ± 0.2	<0.001
Trunk muscle	22.9 ± 2.8	23.8 ± 2.5	19.9 ± 1.9	<0.001
Predicted muscle	6.8 ± 0.8	7.0 ± 0.8	5.9 ± 0.3	<0.001
HGS	16.3 ± 4.4	17.1 ± 4.3	13.4 ± 3.4	<0.001
TUG	15.6 ± 3.9	15.5 ± 4.1	16.0 ± 3.4	0.53
**Biochemistry**				
Glucose (mmol/L)	10.9 ± 4.0	10.9 ± 3.8	11.0 ± 4.6	0.98
HDL-cholesterol (mmol/L)	1.5 ± 0.4	1.5 ± 0.4	1.4 ± 0.4	0.76
Total cholesterol (mmol/L)	5.2 ± 1.1	5.2 ± 1.1	5.3 ± 1.1	0.78
25(OH)D # (nmol/L)	54.6 (39.9–75.9)	54.4 (40.9–75.6)	55.5 (34.6–91.7)	0.35
Irisin (ng/L)	169.1 ± 40.2	180.8 ± 44.3	145.8 ± 11.6	0.001

**Note**: Data presented as mean ± standard deviation; #denotes non-normal distribution and presented as median (inter-quartile range); BMI, body mass index; WHR, waist–hip ratio, MAC, mid-arm circumference; TSF, triceps skinfold-thickness; CI, conicity index; MAMA, mid-arm muscle area; AVI, abdominal volume index; HGS, hand grip strength; TUG, timed up-and-go test; *p*-value significant at <0.05.

**Table 3:** Associations of select parameters with sarcopenia.

Parameters	OR (95% CI)	*p*-Value
**Anthropometrics**		
BMI (kg/m^2^)	0.79 (0.71–0.89)	<0.001
Waist circumference (cm)	0.91 (0.86–0.96)	<0.001
Hip circumference (cm)	0.91 (0.86–0.96)	<0.001
WHR	0.50 (0.001–2.6)	0.82
MAC	0.75 (0.64–0.87)	<0.001
TSF	0.91 (0.80–1.04)	0.16
CI	0.21 (0.002–16.9)	0.48
MAMA	0.90 (0.85–0.95)	<0.001
AVI	0.79 (0.69–0.91)	0.001
**Biochemistry**		
Total cholesterol (mmol/L)	1.07 (0.67–1.72)	0.78
HDL-cholesterol (mmol/L)	0.80 (0.19–3.24)	0.76
Glucose (mmol/L)	1.0 (0.88–1.14)	0.98
25(OH) D (nmol/L)	1.28 (0.14–12.2)	0.83
Irisin (ng/l)	0.97 (0.95–0.99)	0.002
**Macronutrients**		
Total calories (kcal)	1.0 (0.99–1.01)	0.86
Fats (kcal)	1.0 (0.99–1.02)	0.70
Protein (g)	0.99 (0.97–1.03)	0.93
Carbohydrate (g)	0.99 (0.99–1.01)	0.69
Total fiber (g)	0.94 (0.88–0.99)	0.03

**Note:** Data presented as odds ratio (OR); 95% confidence interval (95% CI); BMI, body mass index; WHR, waist–hip ratio; MAC, mid-arm circumference; TSF, triceps skinfold-thickness; CI, conicity index; MAMA, mid-arm muscle area; AVI, abdominal volume index; significance at *p* < 0.05.

## Data Availability

Data are available upon reasonable request to the corresponding author.

## Supplementary Materials

The following supporting information can be downloaded at: https://www.mdpi.com/article/10.3390/nu14091855/s1, Table S1 Physical activity according to sarcopenia status; Table S2 Lifestyle patterns (Sleep and Sun exposure) according to sarcopenia status.
