# Supplementary material for: Screening for Sarcopenia among Elderly Arab Females: Influence of Body Composition, Lifestyle, Irisin, and Vitamin D
Source: Nutrients. 2022 Apr 29;14(9):1855. doi: 10.3390/nu14091855 (PMC9099718; doi:10.3390/nu14091855)
Supplement: Supplementary file 1 [file nutrients-14-01855-s001.zip › nutrients-1666667 - Supplementary Materials version 2.pdf]

Screening for Sarcopenia among Elderly Arab Females: Influence of Body Composition, Lifestyle, Irisin, and Vitamin D

Tafany A. Alsaawi [1], Dara Aldisi [1], Mahmoud M. A. Abulmeaty [1], Malak N. K. Khattak [2], Abdullah M. Alnaami [2], Shaun Sabico [2] and Nasser M. Al-Daghri [2,*]

Table S1. Physical activity according to sarcopenia status.

	All	Non-Sarcopenia	Sarcopenia	*P*-Value
**Light physical activity**				
Eight or more times a week	0 (0.0)	0 (0.0)	0 (0.0)	0.69
Four to seven times a week	18 (13.6)	15 (14.2)	3 (11.5)	
One to three times a week	7 (6.1)	5 (5.7)	2 (7.7)	
Less than one time a week	4 (3.1)	4 (3.8)	0 (0.0)	
I don't exercise	102 (77.3)	81 (76.4)	21 (80.8)	
**Moderate Physical Activity**				
Eight or more times a week	0 (0.0)	0 (0.0)	0 (0.0)	0.37
Four to seven times a week	18 (13.6)	16 (4.7)	2 (7.7)	
One to three times a week	5 (3.8)	5 (4.7)	0 (0.0)	
Less than one time a week	2 (1.5)	2 (1.9)	0 (0.0)	
I don't exercise	106 (81.1)	82 (78.3)	24 (92.3)	
**Vigorous Physical Activity**				
I don't exercise	131 (100)	105 (100.0)	26 (100)	1.0

Note: Data presented N (%). *P*-value significant at < 0.05.

Table S2. Lifestyle patterns (Sleep and Sun Exposure) according to sarcopenia status.

	All	Non-Sarcopenia	Sarcopenia	*P*-Value
**How many hours on average do you sleep at night?**				
Less than 2 hours	0 (0.0)	0 (0.0)	0 (0.0)	0.82
2–4 hours	37 (28.2)	30 (28.6)	7 (26.9)	
5–7 hours	69 (52.7)	54 (51.4)	15 (57.7)	
8 hours or more	25 (19.1)	21 (20.0)	4 (15.4)	
**What time do you usually sleep at night?**				
Any time between 8 to 9:50	12 (9.2)	9 (8.6)	3 (11.5)	0.69
Any time between 10 to 11:50	41 (31.3)	34 (32.4)	7 (26.9)	
Any time between 12 to 1:50	61 (46.6)	47 (44.8)	14 (53.8)	
Any time between 2 to 4	17 (13.0)	15 (14.3)	2 (7.7)	
**What time do you wake up in the morning?**				
Before 7 a.m.	72 (55.0)	56 (53.3)	16 (61.5)	0.26
Any time between 7 to 9	56 (42.7)	47 (44.8)	9 (34.6)	
Any time between 9:10 to 11	1 (0.8)	0 (0.0)	1 (3.8)	
Any time between 11:10 to 1	1 (0.8)	1 (1.0)	0 (0.0)	
Any time between 1:10 to 3	1 (0.8)	1 (1.0)	0 (0.0)	
**Average time in bed before falling asleep?**				
0–15 minutes	70 (53.4)	58 (55.2)	12 (46.2)	0.44
16–30 minutes	34 (26.0)	24 (22.9)	10 (38.5)	
31–45 minutes	5 (3.8)	4 (3.8)	1 (3.8)	
46–60 minutes	16 (12.2)	13 (12.4)	3 (11.5)	

more than 60 minutes	6 (4.6)	6 (5.7)	0 (0.0)	
**Do you sleep in the middle of the day (nap)?**				
Always	20 (15.3)	16 (15.2)	4 (15.4)	0.22
Often	22 (16.8)	14 (13.3)	8 (30.8)	
Sometimes	30 (22.9)	24 (22.9)	6 (23.1)	
Rarely	4 (3.1)	4 (3.8)	0 (0.0)	
Never	55 (42.0)	47 (44.8)	8 (30.8)	
**If you sleep during the daytime (nap)—for how long?**				
Less than 1 hour	27 (37.0)	20 (36.4)	7 (38.9)	0.71
1–2 hours	44 (60.3)	33 (60.0)	11 (61.1)	
More than 2 hours	2 (2.7)	2 (3.6)	0 (0.0)	
**Exposed to the sun during the past week**				
Less than 5 minutes daily	4 (3.1)	3 (2.9)	1 (3.8)	0.78
(5–15) minutes daily	18 (13.7)	15 (14.3)	3 (11.5)	
(15–30) minutes daily	7 (5.3)	6 (5.7)	1 (3.8)	
More than 30 minutes daily	5 (3.8)	5 (4.8)	0 (0.0)	
I don't expose myself to the sun	97 (74.0)	76 (72.4)	21 (80.8)	
**When do you usually expose yourself to the sun?**				
Right after sunrise to 10 A.M.	5 (3.8)	5 (4.8)	0 (0.0)	0.57
From 10 a.m. to 12 p.m.	11 (8.4)	9 (8.6)	2 (7.7)	
From after 10 a.m. to 3 p.m.	7 (5.3)	7 (6.7)	0 (0.0)	
From after 3 p.m. to sunset	5 (3.8)	4 (3.8)	1 (3.8)	
No specific time	6 (4.6)	4 (3.8)	2 (7.7)	
I don't expose myself to the sun	97 (74.0)	76 (72.4)	21 (80.8)	
**Expose your body to the sun's rays directly**				
Yes	32 (24.4)	27 (25.7)	5 (19.2)	0.68
No	3 (2.3)	2 (1.9)	1 (3.8)	
I don't expose myself to the sun	96 (73.3)	76 (72.4)	20 (76.9)	

Note: Data presented N (%). P-value significant at <0.05.
